# Serum TNF-*α* Level as a Possible Predictor of Inhibitor Levels in Severe Hemophilia A

**DOI:** 10.1155/2021/6483490

**Published:** 2021-11-05

**Authors:** Susi Susanah, Harry Raspati, Nur Melani Sari, Lulu Eva Rakhmilla, Yunia Sribudiani, Octawyana Moestopo, Puspasari Sinaga, Ponpon Idjradinata, Ani Melani Maskoen

**Affiliations:** ^1^Department of Child Health, Hematology-Oncology Division, Dr. Hasan Sadikin General Hospital/Faculty of Medicine, Universitas Padjadjaran, Bandung 40161, Indonesia; ^2^Department of Public Health, Epidemiology and Biostatistic Division, Faculty of Medicine, Universitas Padjadjaran, Bandung 40161, Indonesia; ^3^Department of Biomedical Sciences, Biochemistry and Molecular Biology Division, Faculty of Medicine, Universitas Padjadjaran, Bandung 40161, Indonesia; ^4^Study Center of Medical Genetics, Faculty of Medicine, Universitas Padjadjaran, Bandung 40161, Indonesia; ^5^Faculty of Medicine, Universitas Padjadjaran, Bandung 40161, Indonesia

## Abstract

**Background:**

The development of factor VIII (FVIII) inhibitor in patients with hemophilia A (PWHA) is a great challenge for hemophilia care. Both genetic and environmental factors led to complications in PWHA. The development of inhibitory antibodies is usually induced by the immune response. Tumor necrosis factor *α* (TNF-*α*), one of the cytokines, might contribute to its polymorphism. In this study, we investigated the clinical factors, level of serum TNF-*α*, and polymorphism of c.−308G > A TNF − *α* gene in inhibitor development in severe PWHA.

**Methods:**

A cross-sectional study was conducted among all PWHA in West Java province. The clinical parameters, FVIII, FVIII inhibitor, and serum TNF-*α* level were assessed. The genotyping of −380G > A TNF-*α* gene polymorphism was performed using polymerase chain reaction and Sanger sequencing.

**Results:**

Among the 258 PWHA, 216 (83.7%) were identified as severe PWHA. The FVIII inhibitor was identified in 90/216 (41.6%) of severe PWHA, consisting of 45 high-titer inhibitors (HTI) and 45 low-titer inhibitors (LTI). There was a significant correlation between serum TNF-*α* level and the development of HTI (*p* = 0.043). The cutoff point of serum TNF-*α* level, which can be used to differentiate between HTI and LTI, was 11.45 pg/mL. The frequency of FVIII replacement therapy was significant only in HTI of severe PWHA regarding serum TNF-*α* level (*p* = 0.028). There is no correlation between polymorphisms of −380G > A TNF-*α* gene and inhibitor development (*p* = 0.645).

**Conclusions:**

The prevalence of FVIII inhibitor in severe PWHA in West Java, Indonesia, was 41.6%. The frequency of replacement therapy is a risk factor for inhibitor development. Serum TNF-*α* level might be used to differentiate between high and low inhibitor levels in severe hemophilia A, and this might support decision making regarding treatment options for inhibitor in severe hemophilia A.

## 1. Introduction

Hemophilia A, the most frequent hereditary bleeding disorder, is an X-linked bleeding disorder due to deficiency in coagulation factor VIII (FVIII) that affects one individual in 5.000–10.000 newborn males [1]. Iorio et al. reported the prevalence (per 100.000 males) is 17.1 cases for all severities of hemophilia A [2]. Hemophilia A comprised 85% of all types of hemophilia, and it is classified as mild (>5%-40%), moderate (1%-5%), and severe (<1%) hemophilia A based on FVIII levels. Patients with hemophilia A (PWHA) need FVIII replacement therapy to stop acute bleeding and maintain hemostasis for their quality of life [1, 3].

The development of an immunogenic response that is characterized by the appearance of alloantibodies called inhibitors against the therapeutic FVIII infused to the patient causes its neutralizing effect of treatment [1, 4]. It is the most relevant adverse event in hemophilia treatment and becomes a major complication of the disease, especially in severe PWHA [3–7]. FVIII inhibitors bind to functional epitopes that are most commonly found in A2, C1, and C2 domains of the factor protein. This binding interferes with the function of infused FVIII. FVIII inhibitors in PWHA are mainly immunoglobulin G (IgG) antibodies of the IgG1 and IgG4 subclass. IgG4 antibodies predominate in patients with high-titer inhibitor (HTI) while IgG1 antibodies are more abundant in patients with low-titer inhibitors (LTI) [7].

The prevalence of inhibitor FVIII in severe PWHA varies from 25% to 30%, and it is less often in those with mild/moderate disease and involved multifactorial factors, such as genetic and environmental factors [1, 5–7]. Previous studies reported different results, and some studies found genetic factors predominate in inhibitor development such as genetic mutation, race/ethnicity, and immunological factors. The type of mutation has been known to affect not only on the degree of severity but also on the risk of inhibitor development in PWHA [1, 6, 7]. Null mutations and large rearrangements of the factor VIII gene appear to confer a higher risk of developing inhibitors compared with point mutations and small insertions/deletions [7, 8]. Other genetic risk factors include family history and genetic variants such as polymorphic immune regulatory gene [7].

Furthermore, individual immune response properties may also affect a patient's reaction to exogenous FVIII, which include certain major histocompatibility complex (MHC) class II system of cytokines and their polymorphisms [8]. Tumor necrosis factor *α* (TNF-*α*) is considered an important cytokine with potent proinflammatory and immunomodulatory functions associated with autoimmune antibody-mediated diseases included in FVIII inhibitor development. Some studies found polymorphisms of several cytokine genes are risk factors for inhibitor development in PWHA. The TNF-*α* −308GA polymorphism located in the promoter regions is considered the most extensively studied polymorphism with pathophysiologic effects [8, 9]. Environmental factors, as potentially modifiable risk factors, including age of first exposure (treatment), types of FVIII replacement, frequency of therapy, and trauma/surgery were associated with inhibitor development [7, 10]. It seems the multiple genetic and environmental factors interact dynamically in inhibitor development [7]. This study is aimed at analyzing the risk factors of FVIII inhibitor development in severe hemophilia A in the population of West Java, Indonesia.

## 2. Materials and Methods

### 2.1. Patient Enrollment and Subject Collection

**Figure 1:**
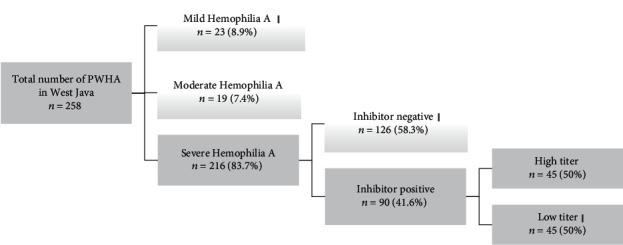
Patient enrollment profile in the study.

This cross-sectional study included all PWHA (258 patients) who registered at West Java Hemophilia A Registry until 2016 (Figure 1). Patient demographic information, their current FVIII level, and their severity of hemophilia classification were collected from the database of West Java Indonesian Hemophilia Society.

Written informed consent for participation in the study was obtained from patients; and for those whose age is <12 years, the consent was obtained from their parents.

### 2.2. Ethical Approval

Ethical approval was obtained from the Ethical Committee of Dr. Hasan Sadikin General Hospital/Faculty of Medicine, Universitas Padjadjaran, Bandung, Indonesia. This study was conducted in accordance with the principles of the Declaration of Helsinki.

### 2.3. Subjects' Characteristics and Data Collection

The clinical parameters of all subjects were assessed to determine age, ethnicity, bleeding episode, age at first therapy, frequency of therapy, and type of FVIII replacement therapy. Data were obtained from patient's medical records and the Indonesian Hemophilia Registry branch of West Java. When this study was conducted, all of the PWHA who classified as severe hemophilia A were in healthy condition, and no signs and symptoms of infection were detected. Peripheral venous blood samples were collected, and a routine hematology test was performed. They were reexamined for the F VIII level to confirm the level regarding the research inclusion at the same time with inhibitor and serum TNF-*α* level, also blood sample for polymorphism of −308G > A TNF-*α* gene test. FVIII and FVIII inhibitor levels were measured using Sysmex CS 2500, and serum TNF-*α* was measured using the ELISA method (Cloud-Clone Corps, USA). Bethesda assay was used to measure the presence of FVIII inhibitor. FVIII level < 1% was classified as severe hemophilia A, inhibitor level < 5 Bethesda unit (BU) was defined as low titer, and inhibitor level ≥ 5 BU was defined as high titer. All of the measurements were performed in the Clinical Pathology Laboratory of Dr. Hasan Sadikin General Hospital.

### 2.4. Genotyping of c.−380G > A Polymorphism

To investigate the polymorphisms of −308G > A TNF-*α* gene in subjects, PCR and DNA sequencing were performed in the Molecular Genetics Laboratory of Faculty of Medicine, Universitas Padjadjaran.

### 2.5. Statistical Analysis

Data analyses were performed using SPSS software version 23. Nongenetic risk factor data, including bleeding episode, age at first therapy, frequency of therapy, and product type of VIII replacement therapy, were evaluated using the Kolmogorov–Smirnov test and chi-square test. There was an association between the presence of inhibitor and the serum TNF-*α* level and polymorphism of −308G > A TNF-*α* gene using independent *t*-test or Mann–Whitney test. The correlation between serum TNF-*α* level and inhibitors was stratified by bleeding episode, age of first therapy, and frequency of therapy using the Mann–Whitney test. A *p* value of < 0.05 was considered statistically significant.

## 3. Results

The data of 258 PWHA in West Java province were collected from the registry: 216 (83.7%) severe PWHA, 19 (7.4%) moderate PWHA, and 23 (8.9%) mild PWHA. Among the 216 severe PWHA, 90 (41.6%) patients expressed FVIII inhibitor. The mean FVIII inhibitor level was 4.79 (2.00) BU/mL with a range of 0.90-11.04 BU/mL. Forty-five patients with HTI had a mean value of 6.46 (1.24) BU/mL, and its range was 5.04-11.04 BU/mL, whereas the mean of other 45 patients with LTI was 3.13 (0.95) BU/mL with a range of 0.90-4.96 BU/mL.  Table 1 presents the nongenetic (environmental) characteristics of severe PWHA with inhibitor.

**Figure 2:**
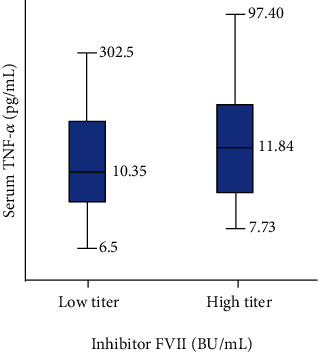
Comparison of serum TNF-*α* and inhibitor FVIII. The median serum TNF-*α* level in LTI was 10.35 pg/mL, range 6.5–302.5 pg/mL, and CI 95%(lower–upper bound) = 4.80–31.02 pg/mL. The median serum TNF-*α* level in HTI was 11.84 pg/mL, range 7.73–97.40 pg/mL, and CI 95%(lower–upper bound) = 11.34–20.20 pg/mL. *p* value = 0.043.

In this study, the median serum TNF-*α* level that widely varies with a range of 6.5-302.5 pg/mL was 11.34 pg/mL.  Figure 2 shows the comparison between serum TNF-*α* levels between two groups of inhibitors, and it is significantly different (*p* = 0.043). On the basis of Spearman's correlation analysis, there was no significant correlation between serum TNF-*α* level and inhibitor (*p* = 0.140).

**Figure 3:**
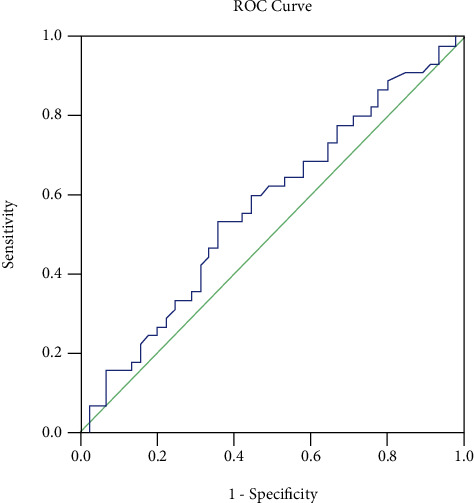
Receiver operating characteristic curve serum TNF-*α* level. The cutoff point of serum TNF-*α* level used as a predictor of HTI was 11.45 pg/mL; sensitivity and specificity were 0.467 and 0.533, respectively; and CI 95%(lower–upper bound) = 0.453–0.690. *p* value = 0.572.

On the basis of the receiver operating characteristic curve, the cutoff point of serum TNF-*α* level used as a predictor of HTI was 11.45 pg/mL (Figure 3).

In both LTI and HTI groups, bleeding episodes had a higher median TNF-*α* level for frequency ≥ 2 times/month, 11.35 (range 8-302.5) pg/mL versus 12.81 (range 7.73-97.4) pg/mL, respectively. TNF-*α* level based on age of the start of therapy for <12 months had a higher median in the HTI group than in the LTI group, 14.05 (range 10.03-18.8) pg/mL versus 9.29 (range 8.56-15.9) pg/mL, respectively. Likewise, TNF-*α* level for frequency ≥ 2 times/month in FVIII frequency replacement therapy had a higher median in the HTI group than in the LTI group, 13.10 (range 7.73–97.4) pg/mL versus 11.25 (range 8–302.5) pg/mL, respectively. On the basis of two clinical variables, bleeding episodes and age of the start of therapy, the correlation between TNF-*α* level and FVIII inhibitor was not statistically significant. Meanwhile, the correlation between TNF-*α* levels in the HTI group was influenced by FVIII frequency replacement therapy (*p* < 0.05) (Table 2).

**Figure 4:**
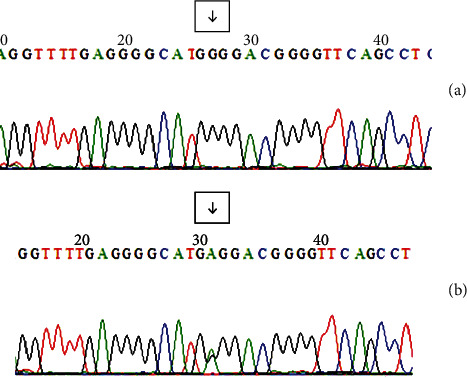
Electropherogram of DNA sequencing polymorphisms of −308G > A TNF-*α*. (a) Arrow indicates site of GG genotype (homozygous wild type). (b) Arrow indicates site of AG genotype (heterozygous).

The assessment of PCR and DNA sequencing polymorphism of −308G > A TNF-*α* gene was demonstrated in Figure 4. The polymorphism location is marked by an arrow. Normal genotype was shown in Figure 4(a), and polymorphism genotype is shown in Figure 4(b). In normal genotype, the peak curve was in (GG), whereas in polymorphism genotype, it was in GA, which figures out the location of polymorphisms of −308G > A TNF-*α* gene.

Furthermore, 85 subjects (94%) had normal genotype (guanosine–guanosine (GG)), 43 (47.8%) subjects had LTI, and 42 (46.7%) subjects had HTI. However, only 5 subjects had polymorphism genotype (guanosine–adenine (GA)), 2 subjects with LTI, and 3 subjects with HTI (*p* = 0.645). On the basis of an independent *t*-test, there was no significant difference between polymorphisms of −308G > A TNF-*α* gene and inhibitor (*p* = 0.645) (Table 3).

## 4. Discussion

West Java is the province with the biggest population in Indonesia, including almost 18% of the total population (46 million of 261 million inhabitants). In this study, the prevalence of severe hemophilia A with inhibitor in West Java was 41.6% with a range of inhibitor level being 0.9-11.4 BU/mL. It is reported that the prevalence of inhibitor development (from all over the world) might vary between 3.6% and 52% based on ethnicity [11]. A previous study that published data in this regard from Indonesia with different ethnicities showed almost the same result of 37.5% in Jakarta and 37.9% in East Java, whereas the results from other regions were not published [12, 13]. Study in Canada reported that inhibitor severe PWHA was 25.9%, while other studies in difference countries from Asian region reported such as Saudi Arabia (29.3%), Japan (26.8%), Egypt (18.2%), Jordan (14.5%), Taiwan (10.4%), India (9.6%), and China (1.4%), respectively [14–21].

The incidence of inhibitor formation varies on the basis of ethnicity with higher rates found among African-American, Latino, and Hispanic patients than among Caucasian patients [1, 11, 22]. In this study, almost 90% of the subjects were Sundanese, as the major ethnic group in West Java province. The association between this ethnic and inhibitor development was not statistically significant. This shows that inhibitor development is not associated with genetic background [1].

The subject's ABO blood group distribution that developed LTI and HTI groups in this study mostly was A and O (as seen in Table 1). Franchini et al. in their study reported that, among 209 severe PWHA, 56 subjects (26.8%) developed inhibitors with 44 subjects (78.6%) among them having HTI [23]. Franchini et al. stated that blood group O had a 45% reduced risk of synthesizing inhibitor against exogenous FVIII and even reduced up to 60% for HTI rather than nonblood group O. The underlying mechanisms might be explained because of blood group type related to half-life and clearance of circulating von Willebrand factor- (VWF-) FVIII complex [23]. In plasma, FVIII had a tight noncovalent complex with VWF that is important for maintaining appropriate plasma levels of FVIII. Individuals with non-O blood type had VWF level 25% higher than individuals with O blood type, and FVIII–VWF complex in O blood type patients is cleared by low-density lipoprotein receptor-related protein on macrophages more rapidly than in non-O blood type patients. Therefore, FVIII half-life is shorter in patients with hemophilia with O blood type. Additionally, it could affect the endocytosis from dendritic cells and following immune effectors [24]. Other hypotheses also stated that a cross-mimicry between FVIII molecule and ABO antigens could be involved in FVIII inhibitor development [24]. These mechanisms still need further investigation. In this study, no significant differences were observed between blood group types.

Hemarthrosis was the most frequent bleeding location developed in both LTI and HTI groups (34.5% versus 36.7%) but not statistically significant. In this study, another frequent bleeding location that was a combination of hemarthrosis- and hematomas-developed inhibitor. A previous study stated that hemarthrosis is a typical bleeding manifestation found in severe hemophilia, especially in patients receiving no maintenance treatment or prophylactic FVIII treatment [25]. Other previous studies also demonstrated that there was an insignificant correlation between bleeding type or location and inhibitor development [5, 25].

In severe hemophilia A, recurrent clinical and subclinical joint bleeding episodes might occur all the time, gradually leading to irreversible changes such as hemophilic arthropathy (HA) [1, 25]. In spite of numerous studies, the pathophysiology of HA has not been fully elucidated, especially as regards immunopathological mechanisms which are associated with the subclinical and early stage of the disease and to be more precise, with chronic joint inflammation. Among numerous compounds participating in the induction of an inflammatory process in the pathogenesis of HA, cytokines seem to play a leading role. The role of inflammatory and anti-inflammatory cytokines in the pathogenesis of HA with respect to cellular and intracellular signaling pathways is still under investigation [6, 26]. The pathophysiological processes occurring in a joint both in active bleeding episode or silent bleeding due to severe deficiency of FVIII probably highly mediated by interactions within the cytokine network and other inflammatory mediators present in the tissues of affected joint. The most important group controlling the disease seems to be well-known inflammatory cytokines, including IL-1*β*, TNF-*α*, and IL-6. Inflammation and proinflammatory cytokines (e.g., TNF-*α*) might be involved in this pathogenesis [27]. Both active bleeding episode or ongoing silence bleeding due to severe deficiency of FVIII will induce danger signals and release of inflammatory substance, such as TNF-ɑ in synovial fluid and circulation (systemic). Zhang et al. reported a significant TNF-*α* accumulation was found in the hemorrhagic tissues of the injured knee and strong TNF-*α* gene upregulation observed since day 3 up to 30 days after hemarthroses. Elevated TNF-*α* level was found in the synovial fluid and plasma which TNF-*α* level in synovial fluids was 5.2-20 folds higher than in plasma (*p* < 0.05) in PWHA [28].

Most patients reported experiencing inhibitors after ≥2 bleeding episodes. In this study, it did not differ significantly between LTI and HTI groups (38.9% versus 41.1%). Saifudin et al. also reported that bleeding episodes did not affect the inhibitor development in severe PWHA [29]. In this study, 76 subjects developed an inhibitor when they started replacement therapy at the age of >12 months with a nearly similar proportion between LTI and HTI groups (*p* = 0.561). This result is in line with a 25-year case–control study by Maclean et al. in 2011, which showed that there was no correlation between age of therapy and inhibitor development [30]. Santagostino et al. in 2005 also found a similar result in 108 PWHA [31]. However, other studies by Lorenzo et al. have identified that the age of the start of therapy can be a predictor of inhibitor risk [32].

Environmental factors that have been suggested to influence the risk of developing inhibitors include both treatment related (i.e., type of product and dosing regimen) and immune system activating risk factors (so-called “danger” signals—a term that refers to the release of inflammatory substances from damaged tissue) [33]. On-demand treatment which gives FVIII replacement therapy during active bleeding/bleeding episode should stimulate immune system activating risk factors. It has been hypothesized that danger signals generated during a bleed might have an adjuvant effect on the immune response to FVIII in on-demand treatment, increasing the inhibitor risk [27]. FVIII treatment in relation to a bleed potentiates inhibitor development compared to FVIII treatment alone in hemophilia A rat, indicating that bleeding is a potential danger signal. This results support the theory that FVIII replacement therapy concurrent with a bleeding episode increases the inhibitor risk. However, clinical studies indicate the danger signal effect, where the immune response is activated by endogenous or exogenous danger or damage signals present at the time and site of FVIII administration. Alloantibodies neutralizing the hemostatic effect of factor VIII develop in PWHA during replacement therapy [33].

In Indonesia, all of the PWHA only received on-demand replacement therapy with plasma-derived FVIII. Fifty-eight subjects who received plasma-derived FVIII infusion developed more inhibitors, especially in HTI groups (32/58). Furthermore, 32 subjects who received mixed therapy (sometimes receiving recombinant or cryoprecipitate) developed lower level inhibitor (19/32) rather than a high-titer inhibitor (13/32). However, these findings were not statistically correlated. Several previous studies concluded different results. The study by Wight and Paisley and the CANAL cohort study demonstrated that some plasma-derived FVIII products may conclude a lower risk of inhibitor development than recombinant FVIII in previously untreated patients (PUPs) with severe hemophilia [6, 34]. A meta-analysis study performed by Franchini in PUPs with severe hemophilia A treated with plasma-derived versus recombinant FVIII concentrates did not support the hypothesis of a higher risk of inhibitor development associated with the use of recombinant FVIII products compared with those treated with plasma-derived FVIII concentrates [35]. More recently, the results of the Research of Determinants of Inhibitor Development among PUPs with hemophilia study reported a similar risk of inhibitor development in both products [36].

In this study, there was no significant association between the frequency of replacement therapy and FVIII inhibitor development (*p* = 1.000), but in further statistical analysis, there was a correlation between the frequency of therapy ≥ 2 times/month and the HTI level in high serum TNF-*α* levels. The more the frequent exposure to FVIII replacement therapy, the more the risk of FVIII antibody formation, so it is a higher risk for FVIII inhibitor development as reported by Maclean et al. in 2011 [30]. Nevertheless, some previous studies did not show the frequency of therapy that has an impact on inhibitor development [5, 20].

Some studies stated that there was a correlation between family history of hemophilia and inhibitor development, whereas this study showed that there was no correlation between history of hemophilia and inhibitor development in hemophilia A [37, 38].

FVIII inhibitors are mainly the IgG, namely, IgG1 and IgG4 subtype [7, 9]. Their development is a complex immune process with multiple genetic and environmental factors that interact dynamically. The ineffective activation regulating CD4+ cells presumably play a role in the development of FVIII inhibitor. The large protein from exogenous FVIII gets fragmented in endocytic vesicle of antigen-presenting cells and then binds to MHC class II molecules. Then, T-cells recognize the antigen and complete T-cell activation through numbers of signals [39].

Some studies suggested that both cytokine patterns and polymorphisms play crucial roles in inhibitor generation [40]. TNF-*α* is a proinflammatory cytokine that has been associated with antibody-mediated and other pathologies. This study stated that serum TNF-*α* level was statistically different between LTI and HTI groups (*p* = 0.043). The development of inhibitory antibodies against exogenous FVIII is usually considered a Th2 cell-induced immune response, whereas TNF-*α* is primarily linked to Th1 cells. However, the cytokine profile clearly indicates the formation of inhibitors to be a mixed Th1 and Th2 cell response, which further underscores the fact that the level of TNF-*α* may also modulate the immune response to the deficient factor in PWHA [11]. It was suggested that Th1 cells serve as initiators of the immune response to FVIII, with Th2 cells responsible for a strong inhibitor production [41, 42]. Then, Chaves et al. suggest that a more anti-inflammatory/regulatory environment may be responsible for significant inhibitor generation. The global cytokine profile demonstrated in peripheral blood leucocytes suggests a major anti-inflammatory/regulatory pattern in PWHA with anti-FVIII inhibitors; findings were confirmed by the *in vitro* stimuli with FVIII [43]. In a recent paper by Qadura et al., it was suggested that different genetic characteristics predispose to certain cytokine responses, which themselves trigger individualized T-helper cell responses [42].

In some autoimmune diseases, namely, inflammatory bowel diseases and myasthenia gravis, there is an association between polymorphism and increased levels of TNF-*α*. Furthermore, the −308A allele has been associated with increased constitutive and inducible transcription levels and with increased production and secretion of TNF-*α* in patients with autoimmune diseases and healthy controls compared with the −308G allele [8]. Astermark et al. had reported the association between genetic polymorphisms of TNF-*α* with inhibitor development in PWHA [8]. Malmo International Brother Study had characterized the causative factors of FVIII mutation, HLA allele, and four polymorphisms in the TNF-*α* gene (−827C > T, −308G > A, −238A > G, and 670A > G) in 164 PWHA. Inhibitors were identified in 46.9% of the patients with −308G/A heterozygote gene and implied that −308G > A TNF-*α* gene polymorphism in Hap2 is a useful marker in PWHA [8]. Pavlova et al. found that the A allele of the −308C > A polymorphism in TNF-*α* was observed with a higher frequency in the inhibitor cohort than in the noninhibitor cohort that was more pronounced for the homozygous A/A genotype [40]. These findings were quite different from those of our study that found the polymorphisms of the −308G > A TNF-*α* gene was not statistically associated with inhibitor development (*p* = 0.645).

Our studies showed that serum TNF-ɑ level correlated with HTI. Nevertheless, the correlation between −380G > A TNF-*α* polymorphism and inhibitor level was not statistically significant. It is assumed that the high level of serum TNF-ɑ was not associated with role of the −380G > A TNF-*α* gene polymorphism, or single polymorphism of cytokine gene was not enough to influence the inhibitor development. Difference characteristic related genetic (ethnicity, race) background, meanwhile the inhibitor development related to high serum TNF-ɑ level can be induced by other inflammatory substance from damage tissue of the joint. Further studies are required to reinforce these findings. Additionally, there are still many factors that influence the FVIII inhibitor development in severe hemophilia A. It seems that genetic factors predominate in inhibitor development.

### 4.1. Limitations of This Study

Some of the limitations of the current study could be noted. Because of the cost constraints and limited facilities in this study, not all parameters related to FVIII inhibitor development, such as FVIII gene mutation type, other cytokine levels, and their polymorphisms, were assessed. The levels of all parameters were measured once (these could be the involvement of transient inhibitor), and therefore, there were some factors affecting inhibitor development, which could be missed. The biomarker of inflammation was not measured.

## 5. Conclusions

The prevalence of inhibitors in severe PWHA in West Java, Indonesia, was high. This study provides data on the potential of using serum TNF-*α* levels in differentiating between high and low inhibitor levels in severe hemophilia A. This finding seems to support decision making on the treatment option of inhibitor in severe hemophilia A. Meanwhile, the frequency of replacement therapy was significantly different between low- and high-titer FVIII inhibitors regarding serum TNF-*α* levels. It seems that genetic factors predominate in inhibitor development.

## Figures and Tables

**Table 1:** Subjects' characteristics.

No.	Characteristics	Inhibitor FVIII (BU/mL)	
		Low titer (*n* = 45)	High titer (*n* = 45)
1.	Age (years old)		
	Mean	10.31	11.66
	SD	6.91	7.66
	Range	1-31	1-35

2.	Father's ethnicity		
	Sunda	33 (36.7%)	36 (40%)
	Jawa	10 (11.1%)	7 (7.8)
	Minang	1 (1.1%)	1 (1.1)
	Aceh	1 (1.1%)	0
	Batak	0	1 (1.1%)

3.	Mother's ethnicity		
	Sunda	40 (44.5%)	41 (45.6%)
	Jawa	4 (4.4%)	3 (3.3%)
	Minang	1 (1.1%)	0
	Batak	0	1 (1.1%)

4.	Blood group		
	A	19 (21.1%)	13 (14.4%)
	B	13 (14.4%)	9 (10.0%)
	AB	0	6 (6.8%)
	O	13 (14.4%)	17 (18.9%)

5.	Bleeding manifestations location (joint-nonjoint)		
	Joint (hemarthrosis)	31 (34.5%)	33 (36.7%)
	Muscle (hematoma)	2 (2.2%)	5 (5.5%)
	Joint and muscle	11 (12.2%)	7 (7.8%)
	Intracranial hemorrhage	1 (1.1%)	0

6.	Bleeding episode		
	<2 times/month	10 (11.1%)	8 (8.9%)
	≥2 times/month	35 (38.9%)	37 (41.1%)

7.	Age of the start of therapy		
	<12 months old	6 (6.7%)	8 (8.9%)
	≥12 months old	39 (43.3%)	37 (41.1%)

8.	FVIII replacement therapy		
	Plasma-derived	26 (28.9%)	32 (35.6%)
	Mixed (recombinant/cryocypitate)	19 (21.1%)	13 (14.4%)

9.	Frequency FVIII replacement therapy		
	<2 times/month	11 (12.2%)	11 (12.2%)
	≥2 times/month	34 (37.8%)	34 (37.8%)

10.	Family history with hemophilia		
	Exist	29 (32.2%)	26 (28.9%)
	None	16 (17.8%)	19 (21.1%)

^∗^ *p* value for all variable was > 0.05.

**Table 2:** Correlation between serum TNF-*α* level and FVIII inhibitor.

TNF-*α* (pg/mL)	FVIII inhibitor (BU/mL)											
	Low titer						High titer					
	Bleeding episodes		Age of the start of therapy		FVIII frequency replacement therapy		Bleeding episodes		Age of the start of therapy		FVIII frequency replacement therapy	
	a	b	c	d	e	f	a	b	c	d	e	f
Median	10.5	11.4	9.3	11.3	11.2	11.3	10.7	12.8	14.1	11.5	10.0	13.1
Range	6.5-15.4	8.0-302.5	8.6-15.9	8.0-302.5	6.5-15.9	8.0-302.5	8.4-14.4	7.7-97.4	10.0-18.8	7.7-97.4	8.4-4.4	7.7-97.4
*p* value	0.218		0.423		0.441		0.171		0.094		0.028	

a = bleeding episode < 2x/month; b = bleeding episode ≥ 2x/month; c = age of the start of therapy < 12 month old; d = age start therapy ≥ 12 month old; e = FVIII frequency replacement therapy < 2x/month; f = FVIII frequency replacement therapy ≥ 2x.

**Table 3:** Comparison of −308G > A TNF-*α* gene and inhibitor FVIII.

Polymorphism of −308G > A TNF-*α* gene	Inhibitor FVIII (BU/mL)		*p*	OR	CI 95%
	Low titer (*n* = 45)	High titer (*n* = 45)			
GG	43 (47.8%)	42 (46.7%)	0.645	1.536	0.244-9.660
GA	2 (2.2%)	3 (3.3%)			

## Data Availability

The data that support the findings of this study are available from the corresponding author upon reasonable request.
